# Effects of ginger ethanol extract on growth performance, antioxidant capacity and intestinal microbiota of broilers

**DOI:** 10.1016/j.psj.2025.106139

**Published:** 2025-11-20

**Authors:** Kaige Yang, Fuxian Gao, Jiajia Shi, Zhiguang Yue, Sanjun Jin, Ping Wang, Chaoqi Liu, Qingqiang Yin, Xiaowei Dang, Hongwei Guo, Juan Chang

**Affiliations:** a College of Animal Science and Technology, Henan Agricultural University, Zhengzhou 450046, China; b College of Animal Science, South China Agricultural University, Guangzhou 510642, China; c Henan Anjin Biotechnology Co., Ltd., Xinxiang 453000, China; d Henan Delin Biological Product Co., Ltd., Xinxiang 453000, China; e College of Biology and Food Engineering, Huanghuai University, Zhumadian 463000, China

**Keywords:** Ginger ethanol extract, Antioxidation, Broilers, Growth performance, Gut microbiota

## Abstract

Ginger ethanol extract (**GEE**), an oily bioactive compound derived with antioxidant and anti-inflammatory properties that is extracted from ginger through ethanol extraction, remains unexplored in terms of its effect on broiler growth and antioxidant mechanism. This study aimed to optimize the extraction process of GEE and evaluate the effect of various concentrations of GEE on growth performance, antioxidant capacity, and intestinal microbiota in broilers. The optimal extraction conditions were: solid-liquid ratio of 8 mL/g using 75 % ethanol, 1 h extraction time. In this condition, the main compound 6-gingerol in GEE reached 10.01 mg/g. A total of 240 one-day-old white feather 817 broilers were assigned to 4 groups with 6 replicates of 10 chickens each: control (basal diet), 0.1 %, 0.2 % and 0.3 % GEE supplementation, The feeding trial lasted for 42 days. Results showed that 0.1 % GEE significantly increased final body weight, ADG, and ADFI by day 42, although 0.3 % GEE reduced early feed intake, it improved ADFI later (*P* < 0.05). The 0.1 % GEE improved crude protein and phosphorus metabolism, 0.3 % GEE improved phosphorus metabolism (*P* < 0.05). In addition, 0.2 % GEE improved cooked meat rate, and 0.3 % GEE improved meat pH and color (*P* < 0.05). Serum analysis indicated that 0.1 % GEE significantly decreased ALT and AST levels, increased serum CAT and liver SOD activity (*P* < 0.05), which indicate that the GEE enhances the antioxidant capacity and liver protection function. Hematoxylin−Eosin analysis indicated the addition of 0.1 % GEE increased jejunal villus length and reduced abdominal fat cell diameter with (*P* < 0.05). Microbial analysis showed a significant increase in *Firmicutes* and *Faecalibacterium* abundance, while a decrease in *Bacteroidota* in the 0.1 % GEE group (*P* < 0.05). In conclusion, the addition of 0.1 % GEE can improve the growth performance, meat quality, lipid and nutrient metabolism of broilers, and protect liver and intestinal health.

## Introduction

Large-scale intensive poultry farming exposes birds to numerous stressors and disease threats, among which oxidative stress is an important factor contributing to reduced growth performance and increased susceptibility to diseases (Sumanu et al., 2022), This situation has been exacerbated in recent years by extreme climate fluctuations, leading to substantial economic losses (Talukder, et al., 2017), What makes it worse, the prohibition of antibiotics has made it difficult for people to cope with the losses caused by these stress injuries. (Chandrakar, et al., 2023). Consequently, there has been growing interest in recent years in exploring natural plants and their extracts such as glycyrrhiza, ginger, garlic, fructus crataegi, curcumin, suberosin, saffron and essential oils as feed or feed additives to enhance growth performance and antioxidant capacity (Apalowo, et al., 2024; Niknia, et al., 2023; Vakili, et al., 2025a).

Ginger ethanol extract (GEE) is rich in bioactive compounds such as gingerols and zingiberene, and has strong antioxidant and anti-inflammatory effects, which have been confirmed in our previous study on the effect of 6-gingerol in broiler primary hepatocytes and GEE on the production performance and antioxidant capacity of laying hens (Jin, et al., 2025; Yang, et al., 2024), this making it a promising alternative to antibiotic growth promoters (AGPs) in poultry nutrition (An, et al., 2016). Other research indicated that ginger oil can enhance antioxidant capacity and promote the release of specific enzymes, thereby exerting its bioactive functions (Abd El-Hack, et al., 2024). Therefore, GEE helps alleviateate oxidative stress in animals, which is essential for maintaining poultry health and productivity.

Although ginger ethanol extract has potential value, the ethanol extraction process and the composition of active ingredients remain the bottlenecks for its large-scale application, Moreover, the systematic effects of different doses of GEE on the growth performance, antioxidant capacity, and intestinal microbiota of broilers are still unclear, and the potential mechanism of its effects also needs to be revealed. To fill this gap, in this study, based on optimizing the standardized extraction process of GEE, through comprehensive assessment of the effects of different addition concentrations on the growth, antioxidant capacity, and intestinal health of broilers, the biological function and mechanism of action of GEE were deeply revealed.

## Materials and methods

### Extraction, preparation and component determination of GEE

Fresh Shandong ginger was sliced into 2-3 mm pieces and dried at 50 ℃ until a constant weight was achieved. After equilibrating in indoor environment for 24 h, air-dried ginger samples were prepared. The ginger was ground and sieved through a 40-mesh screen prior to extraction. The effects of varying ethanol concentrations, solid-liquid ratios, extraction times, and temperatures on 6-gingerol content were evaluated. 6-Gingerol content in ginger ethanol extract was determined using high-performance liquid chromatography (HPLC), with octadecylsilane-bonded silica gel as the stationary phase. The mobile phase consisted of 40 % acetonitrile, 5 % methanol, and 55 % water. The column temperature was maintained at 30 ℃, with a flow rate of 1 mL/min and a detection wavelength of 282 nm. For the analysis of 6, 8, and 10-gingerol, the mobile phase components were acetonitrile (A) and 0.1 % formic acid (B). The column conditions remained the same, with a flow rate of 0.5 mL/min and a detection wavelength of 282 nm; the gradient elution program is presented in Table S1.

### Preparation of GEE feeding

Ginger ethanol extract was extracted using the optimized alcohol extraction method following the crushing of dried Shandong ginger. The obtained ginger ethanol extract was then mixed with SiO_2_ as a carrier at a ratio of 2:1 to prepare a powdered additive, which was sealed and stored in a cool, dry place and mixed into the basic diet before feeding.

### Experimental birds, diets, and management

The animal experiment was conducted at the experimental breeding base of a farm in Tongxu County, Kaifeng City. A total of 240 one-day-old white feather 817 broilers were randomly assigned to four groups, with six replicates of ten birds per replicate. The experiment was divided into two stages: the early stage (1-21 days) and the later stage (22-42 days). All experimental procedures were carried out in accordance with the guidelines of the Animal Welfare and Ethics Committee of Henan Agricultural University (SKLAB-B-2010-003-01). The treatment groups were as follows:

(A) Control Group: Basal diet
(B) 0.1 % GEE Group: Basal diet + 0.1 % ginger ethanol extract
(C) 0.2 % GEE Group: Basal diet + 0.2 % ginger ethanol extract
(D) 0.3 % GEE Group: Basal diet + 0.3 % ginger ethanol extract

Birds from each group were housed in metal wire cages (1.5 m × 1.0 m × 0.5 m), each equipped with drinkers and feeders that allowed free access to feed and water. The environmental temperature was set at 35 ℃ on day 1 and gradually decreased by 0.5 ℃ per day until reaching 25 ℃. Vaccinations were administered in accordance with the rearing standards for 817 broilers, and lighting conditions were adjusted according to broiler welfare requirements. The dose range of ginger ethanol extract was determined based on previous studies, and all experimental procedures and protocols were approved by the Animal Care Committee of Henan Agricultural University. The composition and nutritional levels of the diets are detailed in Table 1.

**Table 1:** Composition and nutritional levels of experimental diet (%, air-dried basis).

Items	1-21 d	22-42 d
Ingredients		
Corn	62.43	67.40
Soybean meal	30.00	25.24
fish meal	2.00	1.00
Soybean oil	2.00	3.00
CaHPO_4_	1.50	1.40
Limestone	1.23	1.18
Met	0.10	0.11
Lys	0.07	
NaCl	0.37	0.37
Premix	0.30	0.30
Total	100.00	100.00
Nutritional levels		
ME(MJ/kg)	12.43	12.86
CP	20.02	17.96
Ca	0.98	0.88
TP	0.68	0.62
AP	0.41	0.36
Met	0.42	0.40
Lys	1.10	0.89

Note: In addition to ME, Met, Lys and AP, the rest of the nutritional composition were measured values. Premix provides: 12000 IU of vitamin A per kilogram of feed; Vitamin D_3_ 3000 IU; Vitamin E 20 IU; Vitamin K_3_ 1.0 mg; Vitamin B_1_ 2.0 mg; Vitamin B_2_ 6.0 mg; Vitamin B_6_ 3.5 mg; Vitamin B_12_ 0.01 mg; D-biotin 0.15 mg; Folic acid 1.25 mg; Nicotinic acid 35 mg; D-Calcium pantothenate 10 mg; Copper 8.0 mg; Iron 100 mg; Manganese 80 mg; Zinc 60 mg; Iodine 0.45 mg; Selenium 0.35 mg.

### Sample collection

On day 43, Broilers were fasted for 12 h, six birds were selected from each pen (with average BW) were selected to record the live weight. and then they were humanely euthanized for blood collection. Blood samples were collected into coagulation tubes and centrifuged at 3000 rpm for 10 min at 4 ℃ to separate the serum, which was then aliquoted and stored at −20 ℃ for subsequent analysis.

Following slaughter, the liver, cecum, bursa of Fabricius, and breast muscle were completely excised and weighed. Portions of the liver and jejunum tissues were fixed in 4 % paraformaldehyde for paraffin embedding and the preparation of hematoxylin and eosin (H&E) stained sections. Samples of jejunal mucosa and cecal contents were collected in sterile cryopreservation tubes and stored at −80 ℃ for later analysis. Additionally, liver samples measuring approximately 1 cm³ were rapidly frozen in liquid nitrogen and subsequently stored at −80 ℃.

### Growth performance

The body weight of broilers was recorded at 21 d and 42 d, respectively. The feed consumption was recorded weekly, and the average body weight, average daily gain (**ADG**), average daily feed intake (**ADFI**) and feed conversion ratio (**FCR**) were calculated accordingly.

### Nutrient metabolic rates

Broiler excreta were collected over four consecutive days during days 17–20 and 38–41 of the experiment using a total fecal collection method. Feathers, feed, and other debris were removed from the feces before weighing. After sampling, an appropriate amount of 10 % sulfuric acid, and the samples were stored in a refrigerator at −20 ℃. Subsequently, the samples were dried at 65 ℃ to a constant weight and then ground for the determination of various parameters. After equilibrating in indoor environment for 24 h, the contents of crude protein (**CP**), crude fat (**EE**), calcium (**Ca**), and phosphorus (**P**) were analyzed in accordance with the Chinese national standards GB/T 6432-2018, GB/T 6433-2006, GB/T 6436-2018, and GB/T 6437-2018, respectively.

### Slaughter performance

After broilers were slaughtered, the slaughter weight, eviscerated weight, half eviscerated weight, breast muscle weight, and abdominal fat weight, as well as the weights of the bursa of fabricius and liver were recorded. Slaughter rate, full evisceration rate, half evisceration rate, breast muscle rate, abdominal fat rate, and organ indices were calculated. The calculation formula of slaughter performance was made as follows:

$$\text{Slaughter}\;\text{rate}\;\left(\%\right)=\frac{\text{Slaughter}\;\text{weigh}}{\text{Live}\;\text{weight}}100\;\%$$

$$\text{Full}\;\text{evisceration}\;\text{rate}\;\left(\%\right)=\frac{\text{Full}\;\text{evisceration}\;\text{weight}}{\text{Live}\;\text{weight}}100\;\%$$

$$\text{Half}\;\text{evisceration}\;\text{rate}\;\left(\%\right)=\frac{\text{Half}\;\text{evisceration}\;\text{weight}}{\text{live}\;\text{weight}}100\;\%$$

$$\text{Breast}\;\text{muscle}\;\text{rate}\;\left(\%\right)=\frac{\text{Breast}\;\text{muscle}\;\text{weight}}{\text{Full}\;\text{evisceration}\;\text{weight}}100\;\%$$

$$\text{Organ}\;\text{index}\;\left(g/\text{kg}\right)=\frac{\text{Organ}\;\text{weight}}{\text{Live}\;\text{weigh}}$$

### Meat quality

After the broilers were slaughtered, chicken breast meat color (L*, a*, and b* values), pH value, drip loss, cooked meat rate, and shear force were measured at 15 min and 24 h. The pH of three different muscle regions was determined using a portable pH meter (PHS-3C, Insa Scientific Instrument Co., Ltd., China), and the average value was calculated. Meat color from three different muscle regions was assessed using an NS800 spectrophotometer (Sanenshi Technology, Shenzhen, China), and the average value was also calculated. Muscle shear force was measured using a C-LM3 muscle tenderness instrument (Tianxiangfeiyu Technology, Beijing, China). Each sample was tested three times, and the average of the measurements was recorded. Two separate breast muscle samples were used to determine drip loss and cooked meat rate, respectively. The breast muscle was weighed (WD1) and placed in a drip loss tube. After storage at 4 °C for 24 h, surface moisture was removed with filter paper and the sample was reweighed (WD2). Another breast muscle sample was weighed (WC1), boiled at 100 °C for 30 min, cooled for 20 min, surface moisture was absorbed with filter paper, and then reweighed (WC2). The calculation formula of drip loss and cooked meat rate is as follows.

$$\text{Drip}\;\text{loss}\;\left(\%\right)=\frac{\text{WD}1-\text{WD}2}{\text{WD}1}100\;\%$$

$$\text{Cooked}\;\text{meat}\;\text{rate}\;\left(\%\right)=\frac{\text{WC}2}{\text{WC}1}100\;\%$$

### Blood biochemical parameters

The serum levels of various biochemical indicators were measured using an automatic biochemical analyzer (Mindray, Shenzhen, China). The parameters analyzed included alanine aminotransferase (**ALT**), aspartate aminotransferase (**AST**), alkaline phosphatase (**ALP**), lactate dehydrogenase (**LDH**), glucose (**GLU**), total protein (**TP**), albumin (**ALB**), triglycerides (**TG**), total cholesterol (**TC**), high-density lipoprotein cholesterol (**HDL-C**), and low-density lipoprotein cholesterol (**LDL-C**).

### Antioxidant index and serum inflammation, immune index

The isolated liver tissue was homogenized in PBS solution, and the protein concentration of the liver homogenate was determined using a total protein quantitative determination kit. The levels of malondialdehyde (**MDA**), total antioxidant capacity (**T-AOC**), total superoxide dismutase (**T-SOD**), catalase (**CAT**), and glutathione peroxidase (**GSH-Px**) in both serum and liver homogenate were measured using corresponding assay kits. The activities of inflammatory factors, including TNF-α, IL-6, and IL-1β, as well as the concentrations of immunoglobulins IgA, IgM, and IgG in serum, were measured using specific ELISA kits in accordance with the instructions of manufacturer.

### Organ Hematoxylin-Eosin (HE) Staining

Hematoxylin-Eosin staining (HE) was performed on the liver, jejunum, breast muscle and abdominal fat tissues fixed in 4 % formaldehyde solution. Villus height (**VH**) and crypt depth (**CD**) were measured using K-Viewer software (kfbio, Ningbo, China), and VH/CD was calculated. At least six different microscopic fields were selected for each section of muscle and abdominal fat, and Image-Pro Plus 6.0 (Media Cybernetics, Rockville, MD, USA) was used to measure muscle fiber radius and abdominal fat particle size.

### Caecum intestinal microbiota analysis

Microbial diversity in the cecal contents was assessed following the standard protocol provided by Meggen Biotech. Genomic DNA from the in vitro fermentation samples was extracted using the E.Z.N.A.® Soil DNA Extraction Kit (Omega Bio-tek, Norcross, GA, USA) according to the instructions of manufacturer and subjected to 1 % agarose gel electrophoresis for quality verification. DNA concentration and purity were quantified using a NanoDrop 2000 UV-Vis spectrophotometer (Thermo Scientific, Wilmington, DE, USA). The V3-V4 hypervariable region of the 16S rRNA gene was amplified using the primer pair 338F (5′-ACTCCTACGGGAGGCAGCAG-3′) and 806R (5′-GGACTACHVGGGTWTCTAAT-3′). The purified PCR products were sequenced on the Illumina MiSeq PE300 platform (Illumina, San Diego, CA, USA) following the standard protocol provided by Majorbio Bio-Pharm Technology Co. Ltd. (Shanghai, China). All bioinformatics analyses were conducted using the Majorbio Cloud Platform (https://cloud.majorbio.com).

Rarefaction curves and alpha diversity indices were generated using mothur software (http://www.mothur.org/wiki/Calculators) (Schloss, et al., 2009), and differences in alpha diversity between groups were evaluated using the Wilcoxon rank-sum test. Principal Coordinate Analysis (PCoA), based on the Bray-Curtis dissimilarity index, was performed to assess the structural similarity of microbial communities across samples. Bar plots illustrating the composition of microbial communities were generated using R software, and PERMANOVA was applied to detect significant differences in microbial community structures among sample groups. Species were selected for correlation network analysis based on Spearman correlation coefficients (|r| > 0.6, *P* < 0.05) (Barberán, et al., 2012).

### Statistical analyses

The data were expressed as mean ± standard error of the mean (SEM), with the sample size (n) indicated in the corresponding notes. The Shapiro-Wilk test was used to evaluate the normality of the data. For the comparison between the two groups, the unpaired student t-test was used. For the comparison of more than two groups, one-way analysis of variance (ANOVA) was performed, and the significant results were post-tested by LSD. Analysis was performed using SPSS 23.0 software (IBM Corporation, Armonk, NY, USA). All figures were generated using GraphPad Prism 9 (GraphPad Software Inc, San Diego, CA, USA) and Adobe Illustrator 2021 (Adobe, San Jose, CA, USA). Statistically, a *P*-value < 0.05 was considered significant, and *P*-value ≥ 0.05 indicated no significant difference.

## Result and analysis

### Extraction process and active ingredient content of GEE

The effects of varying ethanol concentrations, solid-liquid ratios, extraction times, and temperatures on the content of 6-gingerol extracted from ginger were showed in Tables S2, S3, S4, and S5. As shown in Table S2, 6-gingerol extraction rates were significantly higher at ethanol concentrations of 75 %, 85 %, and 99 % compared to 70 % (*P* < 0.05). Table S3 showed that the extraction rate of 6-gingerol was significantly higher than that of 6, 7 and 9 mL/g when the solid-liquid ratio was 8 mL/g (*P* < 0.05). Table S4 showed that the extraction rate of 6-gingerol was significantly higher than other durations when the extraction time was 1 h (*P* < 0.05). Table S5 showed that temperature (20–60 ℃) had no significant effect on extraction efficiency (*P* > 0.05). Based on these findings, the optimal extraction conditions were determined to be 75 % ethanol, a solid-liquid ratio of 8 mL/g, and 1 h. Table S6 showed that under these conditions, the main active components in ginger ethanol extract were measured, with 6-gingerol reaching 10.01 mg/g, followed by 8-gingerol (1.57 mg/g) and 10-gingerol (1.60 mg/g).

### Effect of GEE on production performance of broilers

The effects of different doses of GEE supplementation on broiler production performance are summarized in Table 2. In the early stage (1-21 days), the ADFI in groups D and E was significantly lower than that in group A (*P* < 0.05). During the later stage (22-42 days), groups B, C, and D showed improvements in final body weight, ADG, and ADFI compared to group A. Throughout the whole stage (1-42 days), group B had significantly higher final body weight, ADG, and ADFI (*P* < 0.05), and group D also showed a significant increase in ADFI compared to group A (*P* < 0.05).

**Table 2:** Effect of GEE supplementation on production performance of broilers.

Groups	A	B	C	D	SEM	*P*-value
1-21 d						
Initial weight (g)	37.92	37.92	38.10	38.12	0.075	0.681
Final weight (g)	488.00	488.17	477.33	488.17	3.446	0.636
ADG (g)	21.43	21.44	20.92	21.43	0.164	0.624
ADFI (g)	31.65^a^	31.37^ab^	31.09^a^	30.81^b^	0.127	0.036
Feed/gain	1.48	1.47	1.49	1.44	0.011	0.128
Mortality (%)	0	0	0	0	0	1.000
21-42 d						
Initial weight (g)	488.00	488.17	477.33	488.17	3.446	0.636
Final weight (g)	1290.13^b^	1353.04^a^	1311.73^ab^	1333.70^ab^	8.102	0.025
ADG (g)	38.20^b^	41.18^a^	39.73^ab^	40.26^ab^	0.373	0.025
ADFI (g)	71.41^b^	77.84^a^	74.11^ab^	77.33^a^	0.764	0.002
Feed/gain	1.87	1.89	1.87	1.92	0.019	0.754
Mortality (%)	0	0	0	0	0	1.000
1-42 d						
Initial weight (g)	37.92	37.92	38.10	38.12	0.075	0.681
Final weight (g)	1290.13^b^	1353.04^a^	1311.73^ab^	1333.70^ab^	8.102	0.025
ADG (g)	29.81^b^	31.31^a^	30.32^b^	30.85^ab^	0.193	0.025
ADFI (g)	51.53^b^	54.61^a^	52.82^ab^	54.02^a^	0.393	0.016
Feed/gain	1.73	1.74	1.74	1.75	0.010	0.872
Mortality (%)	0	0	0	0	0	1.000

ab Means with no common superscript within a column differ significantly (*P* < 0.05). 1 Group A: Basal diet; Group B: 0.1 % GEE; Group C: 0.2 % GEE; Group D: 0.3 % GEE. 2 Each value represents the mean value of 6 replicates per treatment (*n* = 6) 3 SEM: standard error of the means.

### Effects of dietary GEE on nutrient metabolic rate of broilers

The effects of GEE supplementation on nutrient metabolism rates in broilers are shown in Table 3. In the early stage, group B showed significantly higher metabolism rates of CP and P (*P* < 0.05), while group D showed a significant increase in phosphorus metabolism rate compared to group A (*P* < 0.05). In the later stage, both group B and group D showed significantly higher phosphorus metabolism rates than group A (*P* < 0.05).

**Table 3:** Effect of GEE supplementation on nutrient metabolism rate of broilers in early stage (%).

Groups	A	B	C	D	SEM	*P*-value
1-21 d						
EE	71.55	73.13	77.62	75.94	1.157	0.250
CP	63.49^b^	72.18^a^	66.61^ab^	68.14^ab^	1.398	0.153
Ca	64.76	68.94	65.17	66.01	1.328	0.707
P	55.06^b^	65.65^a^	58.14^ab^	63.49^a^	1.444	0.025
22-42 d						
EE	54.86	61.00	57.64	64.61	1.890	0.305
CP	58.30	56.94	55.84	52.96	0.902	0.189
Ca	57.03	57.18	57.35	55.34	1.036	0.920
P	53.18^b^	63.06^a^	60.56^ab^	64.52^a^	1.370	0.008

ab Means with no common superscript within a column differ significantly (*P* < 0.05). 1 Group A: Basal diet; Group B: 0.1 % GEE; Group C: 0.2 % GEE; Group D: 0.3 % GEE. 2 Each value represents the mean value of 6 replicates per treatment (*n* = 6) 3 SEM: standard error of the means.

### Effects of dietary GEE on slaughter performance of broilers

The effects of GEE supplementation on slaughter traits in broilers are shown in Table 4. The half evisceration rate in group D was significantly higher than that in group A (*P* < 0.05).

**Table 4:** Effect of GEE supplementation on the slaughter performance of broilers.

Groups	A	B	C	D	SEM	*P*-value
Slaughter rate (%)	91.67	91.71	91.85	90.96	0.211	0.464
Half evisceration rate (%)	79.99^b^	80.34^ab^	81.10^ab^	81.61^a^	0.225	0.034
Full evisceration rate (%)	68.82^b^	70.54^a^	70.80^a^	70.64^a^	0.312	0.073
Breast muscle rate (%)	18.13	18.95	18.45	19.27	0.037	0.233
Abdominal fat rate (%)	2.51	2.49	2.46	2.47	0.139	0.926
Liver index (g/kg)	1.82	1.86	1.94	2.02	0.050	0.983
Bursal index (g/kg)	2.82	2.64	2.58	2.56	0.338	0.677

ab Means with no common superscript within a column differ significantly (*P* < 0.05). 1 Group A: Basal diet; Group B: 0.1 % GEE; Group C: 0.2 % GEE; Group D: 0.3 % GEE. 2 Each value represents the mean value of 6 replicates per treatment (*n* = 6) 3 SEM: standard error of the means.

### Effects of GEE on meat quality of broilers

The effects of GEE supplementation on broiler meat quality are shown in Table 5, Table 6. As shown in Table 5, compared with group A, the pH_15 min_ and pH_24 h_ post-mortem for the breast meat in group D were significantly higher (*P* < 0.05).

**Table 5:** Effect of GEE supplementation on the quality of broilers breast muscle.

Groups	A	B	C	D	SEM	*P*-value
pH_15 min_	5.82^b^	5.99^ab^	6.05^ab^	6.27^a^	0.063	0.080
pH_24 h_	5.52^b^	5.64^ab^	5.62^ab^	5.65^a^	0.019	0.050
Drip loss/%	3.50	3.44	2.82	3.03	0.165	0.407
Cooked meat rate/%	0.60	0.62	0.63	0.62	0.004	0.156
Shear force/N	15.15	18.29	16.69	17.07	0.745	0.591

ab Means with no common superscript within a column differ significantly (*P* < 0.05). 1 Group A: Basal diet; Group B: 0.1 % GEE; Group C: 0.2 % GEE; Group D: 0.3 % GEE. 2 Each value represents the mean value of 6 replicates per treatment (*n* = 6) 3 SEM: standard error of the means.

**Table 6:** Effect of GEE supplementation on the quality of broilers breast muscle (meat color).

Groups	A	B	C	D	SEM	*P*-value
L_15 min_Value	52.21^a^	50.42^ab^	49.06^ab^	45.66^b^	0.800	0.017
a_15 min_Value	5.26	5.46	5.49	5.52	0.155	0.936
b_15 min_Value	8.53	7.72	7.41	7.45	0.264	0.429
L_24 h_Value	53.46^a^	51.61^ab^	48.55^b^	51.91^ab^	0.681	0.065
a_24 h_Value	5.87	6.02	6.36	5.95	0.147	0.690
b_24 h_Value	8.70	8.37	8.41	8.41	0.198	0.953

ab Means with no common superscript within a column differ significantly (*P* < 0.05). 1 Group A: Basal diet; Group B: 0.1 % GEE; Group C: 0.2 % GEE; Group D: 0.3 % GEE. 2 Each value represents the mean value of 6 replicates per treatment (*n* = 6) 3 SEM: standard error of the means.

As shown in Table 6, compared to group A, the L*_15__min_ value (lightness) and b*_15__min_ value (yellowness) in the other experimental groups showed varying degrees of reduction, with group D having a significantly lower L*_15__min_ value (*P* < 0.05), group C also significantly reduced the L*_24__h_ value compared to group A (*P* < 0.05).

### Effects of GEE on serum biochemical indexes of broilers

As shown in Table 7, compared to group A, group B exhibited significantly lower levels of ALT, AST, ALP, and the inflammatory factors IL-6 and IL-1β (*P* < 0.05), in group C, ALT and TG were significantly reduced (*P* < 0.05). Group D showed a significant increase in AST levels compared to group A (*P* < 0.05), while TG, and IL-1β were significantly lower than those in group A (*P* < 0.05).

**Table 7:** Effect of GEE supplementation on serum biochemical indexes of broilers.

Groups	A	B	C	D	SEM	*P*-value
ALT(U/L)	135.55^a^	111.20^b^	108.60^b^	132.40^a^	3.699	0.002
AST(U/L)	291.41^b^	249.98^c^	277.62^b^	339.79^a^	8.521	<0.001
ALP(U/L)	2512.50^a^	1775.60^b^	2444.67^a^^b^	2399.00^a^^b^	126.147	0.079
TP(U/L)	25.50	26.32	25.53	27.44	0.434	0.362
ALB(g/L)	13.90	14.40	14.03	14.26	0.164	0.716
GLB(g/L)	12.07	11.62	11.27	13.10	0.317	0.194
LDH-L(g/L)	1680.17	1646.67	1428	1488.5	99.552	0.795
GLU(mmol/L)	11.63	11.42	10.57	11.07	0.186	0.210
TCHO(mmol/L)	5.22	4.64	5.04	4.92	0.145	0.566
TG(mmol/L)	0.37^a^	0.32^a^^b^	0.28^b^	0.16^c^	0.020	<0.001
HDLC(U/L)	4.57	4.94	4.60	4.87	0.062	0.068
LDLC(mmol/L)	2.16	2.10	2.12	1.82	0.068	0.249
TNF-α(pg/mL)	98.99	89.87	92.90	93.79	3.633	0.864
IL6(pg/mL)	28.29^a^	23.32^b^	27.85^a^	26.98^a^	0.693	0.033
IL1β(pg/mL)	201.83^a^	177.62^b^	188.07^a^^b^	174.13^b^	4.274	0.085

a-c Means with no common superscript within a column differ significantly (*P* < 0.05). 1 Group A: Basal diet; Group B: 0.1 % GEE; Group C: 0.2 % GEE; Group D: 0.3 % GEE. 2 Each value represents the mean value of 6 replicates per treatment (*n* = 6) 3 SEM: standard error of the means.

### Effects of GEE on antioxidant indexes of broilers

**Fig. 1:**
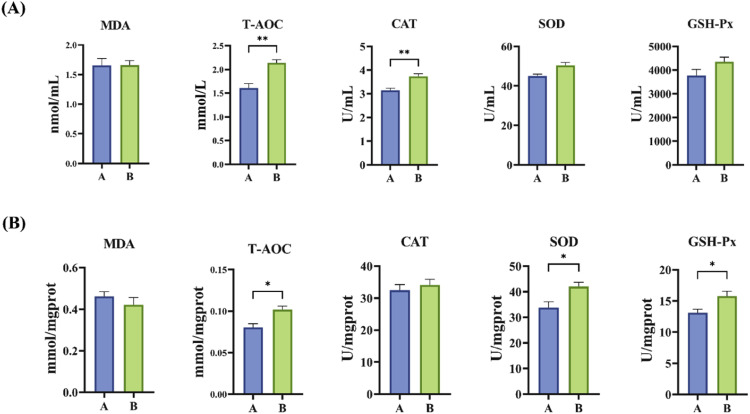
Effect of GEE supplementation on antioxidant indexes of broilers (*n* = 6). “*” indicates *P* < 0.05, “**” indicates *P* < 0.01. (A): Serum antioxidant indexes of broilers. (B): Liver antioxidant indexes of broilers. Group A: Basal diet; Group B: 0.1 % GEE.

The serum and liver antioxidant indicators for group A (control) and group B (0.1 % GEE) are shown in Fig. 1. Group B significantly increased the T-AOC and CAT levels compared to group A (*P* < 0.05). As shown in Fig. 1B, the liver antioxidant indicators showed that group B had significantly higher levels of T-AOC, SOD, and GSH-Px compared to group A (*P* < 0.05).

### Effects of dietary GEE on histomorphology of liver and intestine in broilers

**Fig. 2:**
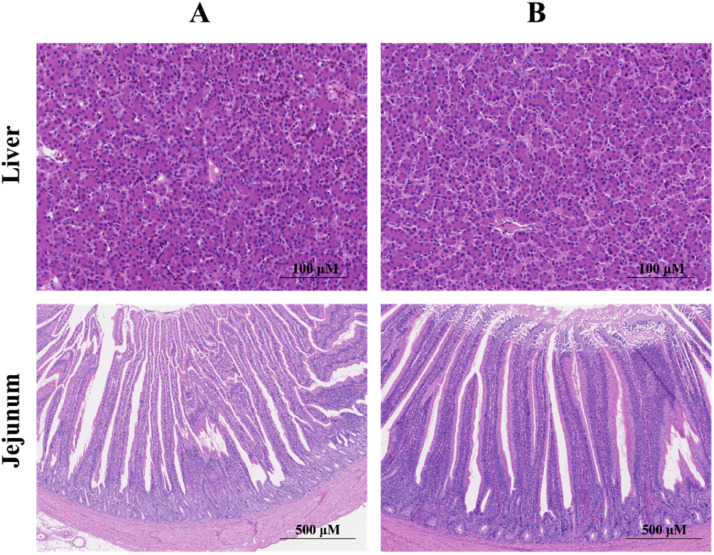
Effect of GEE supplementation histomorphology of liver and intestine in broilers (*n* = 6). Group A: Basal diet; Group B: 0.1 % GEE.

The effects of GEE supplementation on intestinal villus height and crypt depth in broilers are shown in Table 8 and Fig. 2. Group B exhibited a significantly greater villus length compared to group A (*P* < 0.05). Histological analysis of liver tissue via HE staining (Fig. 2) revealed that hepatocytes in all groups were arranged neatly and closely, with intact tissue structure and no obvious pathological changes. In group A, a few cells exhibited mild hemorrhage, whereas group B showed no signs of hemorrhage or necrosis, and the hepatic sinusoids appeared markedly narrower.

**Table 8:** Effects of GEE on villus height and crypt depth in jejunum of broilers.

Groups	A	B	*P*-value
Villus height (µm)	1230.03 ± 24.45^b^	1567.11 ± 36.17^a^	<0.001
Crypt depth (µm)	204.26 ± 7.17^b^	232.24 ± 5.79^a^	0.011
Villus height/Crypt depth	6.06 ± 0.18	6.77 ± 0.22	0.023

ab Means with no common superscript within a column differ significantly (*P* < 0.05). 1 Group A: Basal diet; Group B: 0.1 % GEE. 2 Each value represents the mean value of 6 replicates per treatment (*n* = 6) 3 SEM: standard error of the means.

### Effects of dietary GEE on histomorphology of breast muscle and abdominal fat in broilers

**Fig. 3:**
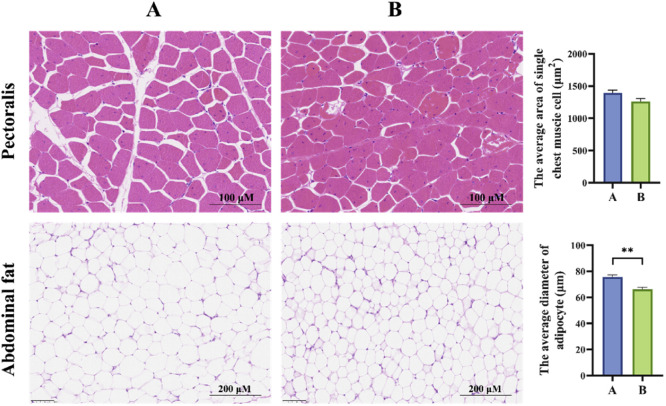
Effect of GEE supplementation histomorphology of pectoralis and abdominal fat in broilers (*n* = 6). “*” indicates *P* < 0.05, “**” indicates *P* < 0.01. Group A: Basal diet; Group B: 0.1 % GEE.

The HE staining results for breast muscle and abdominal fat tissues in broilers are shown in Fig. 3. In group B, muscle fibers exhibited a decreasing trend compared to group A, although the differences were not statistically significant (*P* > 0.05). Additionally, there was a noticeable reduction in the connective tissue between muscle fibers. Group B also showed the diameter of adipocytes in abdominal fat of broilers was significantly less compared to group A (*P* < 0.05).

### Effects of dietary GEE on cecal microflora of broilers

**Fig. 4:**
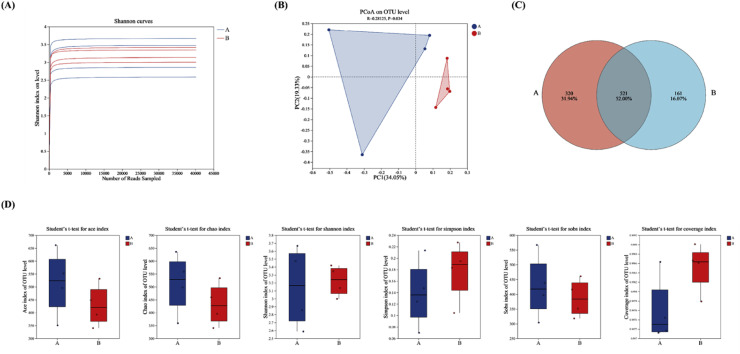
Microbial diversity analysis (*n* = 4). (A) Diluted curve of cecum microflora in each group. (B) PCoA analysis of bacterial flora in cecal contents of broilers. (C) Venn diagram analysis of bacterial flora in cecal contents of broilers. (D) Alpha diversity index of bacterial flora in cecal contents of broilers. Group A: Basal diet; Group B: 0.1 % GEE.

In this study, the 16S rDNA gene V3-V4 region was sequenced from 12 cecal content samples across three treatment groups. OTUs were clustered at a 97 % similarity level, and species annotation, richness, and diversity analyses were conducted. As shown in Fig. 4A the rarefaction curves indicated stabilization and saturation of the sequencing data, confirming comprehensive coverage of species present.

As revealed by the PCoA analysis in Fig. 4B, there were significant differences in cecal microbiota composition among the groups, with Principal Component 1 and Principal Component 2 explaining 34.05 % and 19.33 % of the variance, respectively. The Venn diagram analysis of the cecal microbiota, presented in Fig. 4C, identified a total of 1002 OTUs, with 521 shared among all groups, and 320 and 161 unique OTUs for group A and group B, respectively. As shown in Fig. 4D, alpha diversity analysis revealed no significant differences in ACE, Chao, Shannon, Simpson, Sobs and Coverage indices (*P* > 0.05).

### Analysis of strain composition and difference at phylum level and genus level

**Fig. 5:**
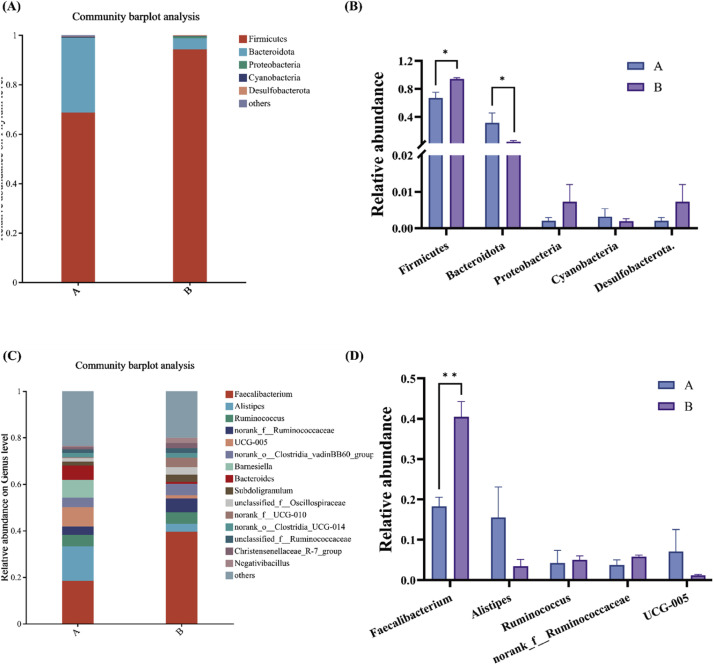
The relative abundance of phylum level and genus level in cecal contents of broilers (*n* = 4). “*” indicates *P* < 0.05, “**” indicates *P* < 0.01. (A) Phylum level species composition of each group. (B) Top 5 Phylum level species abundance difference genus. (C) Genus level species composition of each group. (D) Top 5 Genus level species abundance difference genus. Group A: Basal diet; Group B: 0.1 % GEE.

The microbial composition of cecal contents at the phylum and genus levels is shown in Fig. 5. At the phylum level (Fig. 5A–B), the cecal microbiota of broilers is primarily composed of *Firmicutes, Bacteroidota, Proteobacteria, Cyanobacteria*, and *Desulfobacterota*, among others. Notably, *Firmicutes* and *Bacteroidota* together account for over 90 % of the total phyla, indicating their dominance. The abundance of *Firmicutes* was significantly higher in group B than in group A (*P* < 0.05), whereas the abundance of *Bacteroidota* was significantly lower in group B compared to group A (*P* < 0.05). At the genus level (Fig. 5C–D), the cecal microbiota includes *Faecalibacterium, Alistipes, Ruminococcus, norank_f__Ruminococcaceae, UCG-005, norank_o__Clostridia_vadinBB60_group, Barnesiella, Bacteroides, Subdoligranulum*, and *unclassified_f__Oscillospiraceae*, among others. *Faecalibacterium* is the most abundant genus. Its abundance was significantly higher in group B compared to group A. (*P* < 0.05).

### Correlation analysis of environmental factors and differential microorganisms at genus level

**Fig. 6:**
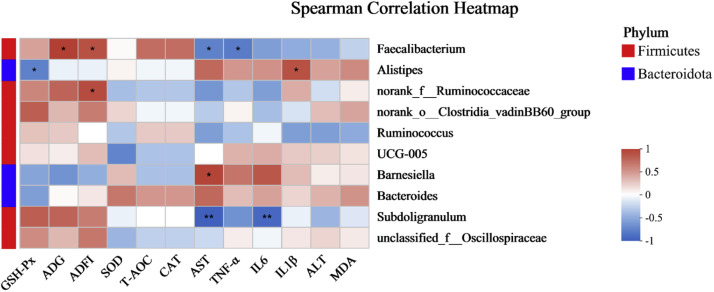
Correlation analysis of microflora with environmental factors in cecal contents of broilers (*n* = 4). The phylum level located in the upper right corner indicated the corresponding phylum level of various bacterial genera in the correlation analysis. Red indicated positive correlations (*r* > 0), and blue indicated negative correlations (*r* < 0). “*” indicates *P* < 0.05, “**” indicates *P* < 0.01.

The relationship between the cecal microbiota at the genus level and environmental factors is shown in Fig. 6. The analysis included the correlation between the top 10 abundant genera and 12 environmental factors, including serum biochemical indicators (ALT, AST, MDA, T-AOC, SOD, CAT, GSH-Px, TNF-α, IL6, IL1β) and whole stage growth indicators (ADG, ADFI).

The results indicated that *Faecalibacterium* was significantly negatively correlated with serum AST and TNF-α (*P* < 0.05), while showing a significant positive correlation with ADG and ADFI (*P* < 0.05). *Alistipes* was significantly negatively correlated with GSH-Px and positively correlated with IL-1β (*P* < 0.05). *Barnesiella* exhibited a significant positive correlation with AST (*P* < 0.05). *Subdoligranulum* showed a significant negative correlation with both AST and IL-6 (*P* < 0.01).

## Disscusion

In recent years, the effect of plant-based feed additives on the production performance of livestock and poultry has attracted considerable attention (Hassan, et al., 2024).Traditional solvents used for extracting plant extracts are primarily lipophilic, such as petroleum ether, methanol, and ethyl acetate. However, taking into account factors related to safety and cost-effectiveness, this study selected ethanol as the preferred extraction solvent. In view of the relatively low boiling point of ethanol, high-temperature processing conditions are not suitable. Therefore, an extraction temperature at room temperature combined with a higher solid-to-liquid ratio was determined to be the most appropriate. Based on these findings, the optimal extraction process for ginger ethanol extract was established.

Through feeding broilers with different concentrations of GEE, it was found that the addition of 0.3 % GEE in broiler diets significantly reduced ADFI without affecting ADG during the early stage, thereby improving FCR. This result is consistent with the research reports of most plant essential oil additives, that is, although the feed intake has decreased, it has little effect on weight gain, thus improving FCR. (Elazab, et al., 2022; Jang, et al., 2007; Lee, et al., 2003; Shanmugavelu, et al., 2004). Some research has indicated that the direct addition of high concentrations of herbal plants to poultry diets may negatively affect sensory quality, leading to reduced palatability and consequently lower feed intake (Hassan, et al., 2024). However, unlike mammals, which often experience aversion to spicy plants due to pain responses, birds are generally more tolerant of such stimuli. For example, pigs exhibit strong aversion to spicy plants (Eisemann and van Heugten, 2007), high doses of rosemary essential oil and ginger essential oil significantly reduced the daily feed intake of New Zealand White rabbits (Elazab, et al., 2022), whereas birds showed minimal reaction to high concentrations of capsaicin and other essential oil mixtures (Mason, et al., 1991; Szolcsányi, et al., 1986). It is worth noting that although 0.3 % GEE in this study reduced the ADFI in the early stage, the recovery of ADFI in the later stage also confirmed the adaptability of broilers to flavor stimulation.

In contrast, the addition of 0.1 % GEE significantly enhanced both average daily gain (ADG) and average daily feed intake (ADFI) during the later stage, suggesting that an optimal level of GEE can improve growth performance. Other studies support this conclusion, indicating that the addition of ginger in poultry feed can increase body weight (Ademola, et al., 2009; Demir, et al., 2003). However, some studies have reported no significant differences in ADFI, FCR and ADG when broilers and quails were fed varying concentrations of ginger essential oil (Herve, et al., 2019; Shanoon, et al., 2012). Additionally, conflicting findings have been documented regarding the effects of ginger root powder or extracts on production performance (Habibi, et al., 2014; Wen, et al., 2020). These discrepancies suggest that, similar to many plant-based additives, variations in research outcomes are not entirely unexpected. They are influenced by multiple factors, including the specific plant source, animal management practices, and whether single or combined spices are utilized (Abdelli, et al., 2021).

The nutrient metabolism rate in poultry reflects the efficiency of digestion, absorption, and utilization of nutrients from feed (Iravani, et al., 2024). In this study, the addition of 0.1 % GEE significantly enhanced the metabolism rates of crude protein (CP) and phosphorus (P) in broilers during the early stage. Furthermore, 0.3 % GEE improved phosphorus metabolism in the early and late stages, indicating that ginger extract had the characteristics of promoting nutrient digestion and utilization, this is consistent with previous reports that gingerol can enhance the digestive ability of broilers. (Wisdom, et al., 2018). The observed increase in CP metabolism with 0.1 % GEE may be attributed to the presence of the proteolytic enzyme zingibain in ginger and the stimulating effect of ginger phenolic components on the secretion of endogenous digestive enzymes. (Al-Khalaifah, et al., 2022). Moreover, the moderate increase of ether extract (EE) metabolism during the late stage of ginger may be due to the fact that ginger can stimulate the production of digestive enzymes such as amylase and lipase (Shewita and Taha, 2018). Overall, the promoting effect of GEE on nutrient metabolism may be due to the fact that the bioactive compounds in ginger stimulated the secretion of digestive enzymes and improved the digestive capacity of poultry (Al-Khalaifah, et al., 2022). On the other hand, The antioxidant and anti-inflammatory properties of ginger ethanol extract, along with its role in promoting gut microbiota homeostasis, further contribute to improved intestinal absorption efficiency (Tekeli, et al., 2010).

The quality of chicken meat significantly influences consumer purchasing decisions, with key indicators including meat color, pH, shear force, drip loss, cooked meat rate, and flavor compounds. pH as an indicator of muscle glycogen hydrolysis ability, reflecting acidity and showing correlation with meat color and tenderness. Consumers frequently associate meat color with freshness, making it a critical quality attribute (Mir, et al., 2017), and it has been proven that supplementing the diet with dietary antioxidants can improve the quality of chicken meat (Vakili and Rashidi, 2011). In this study, 0.3 % GEE significantly increased the pH_15 min_ after slaughter, and decreased the L*_15__min_, while 0.2 % GEE increased the cooked meat rate and decreased the L*_24__h_ value. These changes may be related to the antioxidant properties of GEE. Previous studies have shown that thyme essential oil does not affect chicken breast pH (Gumus and Gelen, 2023), while ginger has been reported to slightly increase meat pH without affecting water-holding capacity (Hassan, et al., 2024). In this study, no significant differences in tenderness were observed among breast meat samples across groups, which is consistent with previous findings on various additives, including ginger, and their effects on meat quality (Li, et al., 2019). Generally, higher L* values are associated with increased oxidation, and an increase in b* values has been attributed to the inherent color of plant extracts, which may influence the results (Gao, et al., 2021).

Serum biochemical indicators provide valuable insights into the metabolic state of animals, nutrient utilization, health status, and organ function. The liver, a key site for biological metabolism, contains various enzymes, particularly transaminases. Damage to liver cells can lead to increased serum levels of ALT and AST, which are specific indicators of liver cell damage, ALP is crucial for phosphate hydrolysis and is present in various tissues, including bone and liver (Hu, et al., 2014). In this study, the addition of 0.1 % GEE significantly reduced the levels of ALT, AST, ALP and inflammatory factors IL-6 and IL-1β in the serum of broilers, indicating that it has the effect of protecting the liver and reducing inflammation., consistent with findings in Japanese quail fed ginger essential oil (Abd El-Hack, et al., 2020). However, higher doses (0.3 % GEE) not only diminished this beneficial effect, but also lead to a rebound in AST levels. Previous studies have shown that low doses of plant active substances can exert beneficial biological functions, while excessive intake may produce potential cytotoxicity and increase liver burden (Hebbar, et al., 2005; Owuor and Kong, 2002). This suggesting that moderate GEE enhances liver function, excessive addition may not be beneficial. Moreover, the addition of 0.2 % GEE significantly lowered serum total cholesterol (TCHO) levels, it further supports the potential of GEE in the regulation of lipid metabolism (Abd El-Hack, et al., 2020). Inflammatory cytokines like IL1β and TNF-α contribute to cell death and tissue damage (Qiao, et al., 2022). This study showed that 0.1 % GEE significantly reduced serum IL6 and IL1β levels, indicated its potential to mitigate inflammatory damage in broilers. This supports previous research that 6-gingerol can inhibit intestinal inflammation through MAPK pathway modulation (Li et al., 2017). The above results indicate that GEE exerts its biological activity through various ways.

Major antioxidant enzymes in animals include CAT, SOD, and GSH-Px. These enzymes function synergistically through various mechanisms to eliminate reactive oxygen species (ROS) and maintain the dynamic equilibrium between oxidation and antioxidation, thus protecting the organism from oxidative damage. In this study, the addition of 0.1 % GEE significantly increased the activities of CAT and SOD in both serum and liver, as well as T-AOC levels in these tissues. This result is partially consistent with the reported results that ginger extract can enhance SOD activity and reduce MDA levels in poultry (An, et al., 2019), it was also consistent with the previous results that GEE increased serum antioxidant enzyme levels and decreased MDA in laying hens (Jin, et al., 2025). However, this study further observed that GEE increased CAT activity and T-AOC level, while GSH-Px activity did not change significantly, suggesting that there may be extract-specific or species-specific differences in the antioxidant pathway of GEE. In summary, GEE can effectively enhance the body 's antioxidant defense function by synergistically regulating a variety of antioxidant enzyme activities and total antioxidant capacity, it indicating that GEE has potential application as an effective natural antioxidant in livestock and poultry production.

Histomorphometry analysis of animal tissues is essential for evaluating organ health, particularly the liver, which plays a central role in metabolism, detoxification, and antioxidant functions. In this study, the addition of 0.1 % GEE maintained intact liver tissue structures, with no significant pathological changes, indicating that GEE has no negative effects on the liver, this is similar to previous research results (Abd El-Hack, et al., 2024). Intestinal villus development is a key indicator of gut health, with villus height positively correlated with nutrient absorption capacity (Vakili, et al., 2025b). In this study, the addition of 0.1 % GEE significantly increased jejunal villus height and improved the villus-to-crypt ratio, suggesting that it could promote the health of intestinal structure, this is similar to previous research (Apalowo, et al., 2024). Other studies have reported similar increases in villus dimensions with GEE (Karangiya, et al., 2016), and feeding oils containing polyphenols has been shown to promote villus height in the duodenum of broilers (Das, et al., 2020). Suggesting that bioactive compounds in ginger, including gingerol, promote gut health and immune regulation. Additionally, the addition of 0.1 % GEE significantly decreased adipocyte diameter, suggesting that GEE enhances fat metabolism in broilers. This finding is consistent with previous reports that ginger supplementation reduces abdominal fat weight (Ademola, et al., 2009). Previous research has indicated that dry ginger can lower respiratory exchange rates and promote fat utilization by increasing fat oxidation. Ginger and its bioactive components exhibit lipid-lowering activity, the mechanism may be related to the inhibition of fat production and enhancement of fatty acid decomposition (Miyamoto, et al., 2015).

Intestinal microbiota is essential for maintaining gut function, aiding in nutrient digestion, immune regulation, and disease defense. The cecum hosts a diverse community of microorganisms that decompose food, maintain mucosal integrity, and promote immune tolerance (Liu, et al., 2024). Feeding is a primary factor influencing the development and function of the gut microbiota (Diaz Carrasco, et al., 2019). Feed composition significantly impacts the gut microbial ecology in chickens, as undigested and unabsorbed feed components provide essential nutrients for microbial growth (Pan and Yu, 2014). At present, various plant feed additives rich in polyphenols and their metabolites have been used to modulate the intestinal microbiota in poultry. In this study, the addition of 0.1 % GEE had no significant effect on the Alpha diversity index of gut microbiota in broilers, but it significantly affected the microbial community structure.

At the phylum level, the cecal microbiota in this study was predominantly composed of *Firmicutes* and *Bacteroidota*, accounting for over 90 % of the total microbial community. *Firmicutes* ferment intestinal contents, producing metabolites such as lactate and propionate, while *Bacteroidota* break down complex carbohydrates, generating beneficial metabolites like butyrate (Fang, et al., 2024; Jordan, et al., 2023; Shin, et al., 2024), they work synergistically, with *Bacteroidota* playing a crucial role in carbohydrate metabolism and gut function (Stubhaug, et al., 2023). The addition of 0.1 % GEE significantly increased *Firmicutes* abundance and reduced *Bacteroidota*, likely due to antibacterial and antioxidant properties of ginger. At the genus level, 0.1 % ginger ethanol extract significantly increased *Faecalibacterium* while moderately decreasing *Alistipes. Faecalibacterium* is important for fermenting dietary fiber into butyrate, which supports gut health and promotes immune regulation (Abd El-Hack, et al., 2022; Lopez-Siles, et al., 2017; Miquel, et al., 2013). Previous studies have indicated that *Faecalibacterium* in the ceca of broilers promotes growth performance (Johnson, et al., 2018), while *Alistipes* contributes to polysaccharide breakdown and gut barrier maintenance (Long, et al., 2024; Su, et al., 2024). Correlation analysis indicated that *Faecalibacterium* was significantly negatively correlated with serum AST, IL1β, and IL6, while positively correlating with average daily gain (ADG) and average daily feed intake (ADFI) in broilers. This suggests that *Faecalibacterium* may help reduce inflammation and protect liver health by modulating the host immune system, with its short-chain fatty acids exhibiting anti-inflammatory effects (AbdEl-Hack, et al., 2022), which may finally enhance gut health, increase feed intake, and promote growth in broilers. In contrast, the proliferation of *Bacteroides* and *Barnesiella*, both from the *Bacteroidota* phylum, may indicated disrupted gut barrier function or heightened inflammatory responses, leading to increased serum AST levels (Zhang, et al., 2023). Previous research has proved that unclassified *Lachnospiraceae* reduces short-chain fatty acid expression in the intestines of mice, thereby promoting inflammatory responses (Huang, et al., 2023).

In this study, we optimized the extraction process of GEE and comprehensively evaluated the effects of GEE on broilers by integrating multiple levels of indicators such as growth performance, antioxidant capacity and gut microbiota. Adding 0.1 % GEE to broiler feed has better antioxidant and growth-promoting effects than higher doses of GEE. High-dose GEE not only reduces palatability and stimulates the gastrointestinal tract, but also increases the liver metabolic burden caused by its own or intermediate metabolites, this is similar to many previously reported results on the effects of high-dose or long-term use of plant active ingredients (Ashrafpour and Ashrafpour, 2025; Elazab, et al., 2022; Kouvedaki, et al., 2024). Therefore, it is of great significance to determine 0.1 % GEE as the optimal dose window.

In conclusion, the addition of 0.1 % GEE can improve the growth performance, meat quality, lipid and nutrient metabolism, the GEE plays an important role in broilers by promoting the antioxidant capacity, alleviating liver damage, and actively regulating the gut microbiota. These findings provide a promising strategy for reducing oxidative stress and improving poultry production.

## CRediT authorship contribution statement

**Kaige Yang:** Writing – original draft, Validation, Methodology, Formal analysis, Data curation. **Fuxian Gao:** Writing – original draft, Validation, Investigation, Formal analysis. **Jiajia Shi:** Methodology, Formal analysis, Data curation. **Zhiguang Yue:** Project administration. **Sanjun Jin:** Software, Methodology. **Ping Wang:** Software, Methodology. **Chaoqi Liu:** Methodology, Funding acquisition. **Qingqiang Yin:** Supervision, Methodology. **Xiaowei Dang:** Project administration, Investigation. **Hongwei Guo:** Writing – review & editing, Methodology. **Juan Chang:** Writing – review & editing, Supervision, Resources.

## Disclosures

The authors declare that there are no conflicts of interest in relation to the manuscript titled "**Effects of Ginger Ethanol Extract on Growth Performance, Antioxidant Capacity and Intestinal Microflora of Broilers**" submitted to “Poultry Science”. We confirm that the results and interpretations reported in the manuscript are original and have not been plagiarized.

The authors declare that the research was conducted in the absence of any commercial or financial relationships that could be construed as a potential conflict of interest.

We certify that we have read and understand the “Poultry Science” conflict of interest policy, All the authors are ultimately responsible and accountable for the contents of the work, we also certify that the AI and AI-assisted technologies were not used in the writing process in this manuscript.

We understand that failure to disclose a conflict of interest may result in the manuscript being rejected or retracted.

## Appendix Supplementary materials

Image, application 1

## Footnotes

Supplementary material associated with this article can be found, in the online version, at doi:10.1016/j.psj.2025.106139.

## References

[bib0001] Abd El-Hack M.E., AboElMaati M..F., Abusudah W.F., Awlya O.F., Almohmadi N.H., Fouad W., Mohamed H.S., Youssef I.M., Al-Gabri N.A., Othman S.I., Allam A.A., Taha A.E., Tellez-Isaias G., Mansour A.M. (2024). Consequences of dietary cinnamon and ginger oils supplementation on blood biochemical parameters, oxidative status, and tissue histomorphology of growing Japanese quails. Poult. Sci..

[bib0002] Abd El-Hack M.E., Alagawany M.., Shaheen H., Samak D., Othman S.I., Allam A.A., Taha A.E., Khafaga A.F., Arif M., Osman A., El Sheikh A.I., Elnesr S.S., Sitohy M. (2020). Ginger and its derivatives as promising alternatives to antibiotics in poultry feed. Anim.: Open Access J. MDPI..

[bib0003] Abd El-Hack M.E., El-Saadony M..T., Alqhtani A.H., Swelum A.A., Salem H.M., Elbestawy A.R., Noreldin A.E., Babalghith A.O., Khafaga A.F., Hassan M.I., El-Tarabily K.A. (2022). The relationship among avian influenza, gut microbiota and chicken immunity: an updated overview. Poult. Sci..

[bib0004] Abdelli N., Solà-Oriol D., Pérez J.F. (2021). Phytogenic feed additives in poultry: achievements, prospective and challenges. Anim.: Open Access J. MDPI.

[bib0005] Ademola S., Farinu G., Babatunde G. (2009). Serum lipid, growth and haematological parameters of broilers fed garlic, ginger and their mixtures. World J. Agric. Sci..

[bib0007] An K., Zhao D., Wang Z., Wu J., Xu Y., Xiao G. (2016). Comparison of different drying methods on Chinese ginger (Zingiber officinale Roscoe): changes in volatiles, chemical profile, antioxidant properties, and microstructure. Food Chem..

[bib0006] Al-Khalaifah H., Al-Nasser A., Al-Surrayai T., Sultan H., Al-Attal D., Al-Kandari R., Al-Saleem H., Al-Holi A., Dashti F. (2022). Effect of ginger powder on production performance, antioxidant status, hematological parameters, digestibility, and plasma cholesterol content in broiler chickens. Anim.: Open Access J. MDPI..

[bib0008] An S., Liu G., Guo X., An Y., Wang R. (2019). Ginger extract enhances antioxidant ability and immunity of layers. Anim. Nutr..

[bib0009] Apalowo O.O., Minor R..C., Adetunji A.O., Ekunseitan D.A., Fasina Y.O. (2024). Effect of ginger root extract on intestinal oxidative status and mucosal morphometrics in broiler chickens. Anim.: Open Access J. MDPI..

[bib0010] Ashrafpour S., Ashrafpour M. (2025). The double-edged sword of nutraceuticals: comprehensive review of protective agents and their hidden risks. Front Nutr..

[bib0011] Barberán A., Bates S.T., Casamayor E.O., Fierer N. (2012). Using network analysis to explore co-occurrence patterns in soil microbial communities. ISME J..

[bib0012] Chandrakar C., Shakya S., Patyal A., Bhonsle D., Pandey A.K. (2023). Detection of antibiotic residues in chicken meat from different agro-climatic zones of Chhattisgarh, India by HPLC-PDA and human exposure assessment and risk characterization. Food Control..

[bib0013] Das Q., Islam M.R., Lepp D., Tang J., Yin X., Mats L., Liu H., Ross K., Kennes Y.M., Yacini H., Warriner K., Marcone M.F., Diarra M.S. (2020). Gut microbiota, blood metabolites, and spleen immunity in broiler chickens fed berry pomaces and phenolic-enriched extractives. Front. Vet. Sci..

[bib0014] Demir E., Sarıca Ş., Özcan M., Suiçmez M. (2003). The use of natural feed additives as alternative to an antibiotic growth promoter in broiler diets. Br. Poult. Sci..

[bib0015] Diaz Carrasco J.M., Casanova N..A., Fernández Miyakawa M.E. (2019). Microbiota, gut health and chicken productivity: what is the connection?. Microorganisms..

[bib0016] Eisemann J.H., van Heugten E. (2007). Response of pigs to dietary inclusion of formic acid and ammonium formate1,2. J. Anim. Sci..

[bib0017] Elazab M.A., Khalifah A..M., Elokil A.A., Elkomy A.E., Rabie M.M., Mansour A.T., Morshedy S.A. (2022). Effect of dietary rosemary and ginger essential oils on the growth performance, feed utilization, meat nutritive value, blood biochemicals, and redox status of growing NZW rabbits. Anim.: Open Access J. MDPI..

[bib0018] Fang Z., Ma M., Wang Y., Dai W., Shang Q., Yu G. (2024). Degradation and fermentation of hyaluronic acid by Bacteroides spp. From the human gut microbiota. Carbohydr. Polym..

[bib0019] Gao Y., Yeh H.-Y., Bowker B., Zhuang H. (2021). Effects of different antioxidants on quality of meat patties treated with in-package cold plasma. Innov. Food Sci. Emerg. Technol..

[bib0020] Gumus R., Gelen S.U. (2023). Effects of dietary thyme and rosemary essential oils on performance parameters with lipid oxidation, water activity, pH, colour and microbial quality of breast and drumstick meats in broiler chickens. Arch. Anim. Breed..

[bib0021] Habibi R., Sadeghi G., Karimi A. (2014). Effect of different concentrations of ginger root powder and its essential oil on growth performance, serum metabolites and antioxidant status in broiler chicks under heat stress. Br. Poult. Sci..

[bib0022] Hassan A.H.A., Youssef I.M.I., Abdel-Atty N.S., Abdel-Daim A.S.A. (2024). Effect of thyme, ginger, and their nano-particles on growth performance, carcass characteristics, meat quality and intestinal bacteriology of broiler chickens. BMC Vet. Res..

[bib0023] Hebbar V., Shen G., Hu R., Kim B.-R., Chen C., Korytko P.J., Crowell J.A., Levine B.S., Kong A.N.T. (2005). Toxicogenomics of resveratrol in rat liver. Life Sci..

[bib0024] Herve T., Raphael K.J., Ferdinand N., Victor Herman N., Willy Marvel N.M., Cyril D’Alex T., Laurine Vitrice F.T. (2019). Effects of ginger (Zingiber officinale, Roscoe) essential oil on growth and laying performances, serum metabolites, and egg yolk antioxidant and cholesterol status in laying Japanese quail. J. Vet. Med. Sci. 2019,.

[bib0025] Hu B., Zou Y., Liu S., Wang J., Zhu J., Li J., Bo L., Deng X. (2014). Salidroside attenuates concanavalin A-induced hepatitis via modulating cytokines secretion and lymphocyte migration in mice. Mediat. Inflamm. 2014,.

[bib0026] Huang Y., Wang Z., Ye B., Ma J.H., Ji S., Sheng W., Ye S., Ou Y., Peng Y., Yang X., Chen J., Tang S. (2023). Sodium butyrate ameliorates diabetic retinopathy in mice via the regulation of gut microbiota and related short-chain fatty acids. J. Transl. Med..

[bib0027] Iravani S., Aziz-Aliabadi F., Vakili R. (2024). Feed processing: a review of the impacts of conditioning time and temperature on feed quality and broilers performance. World's. Poult. Sci. J..

[bib0028] Jang I.S., Ko Y..H., Kang S.Y., Lee C.Y. (2007). Effect of a commercial essential oil on growth performance, digestive enzyme activity and intestinal microflora population in broiler chickens. Anim. Feed Sci. Technol..

[bib0029] Jin S., Shi J., Zhao M., Liu X., Yang K., Shang E., Wang P., Liu C., Wang L., Li X., Yin Q., Yue Z., Dang X., Chang J. (2025). The influence of dietary supplementation with ginger ethanol extract on laying hens' production performance, antioxidant capacity, and gut microbiota. Front. Vet. Sci..

[bib0030] Johnson T.J., Youmans B..P., Noll S., Cardona C., Evans N.P., Karnezos T.P., Ngunjiri J.M., Abundo M.C., Lee C.W. (2018). A consistent and predictable commercial broiler chicken bacterial microbiota in antibiotic-free production displays strong correlations with performance. Appl. Environ. Microbiol..

[bib0031] Jordan C.K.I., Brown R.L., Larkinson M.L.Y., Sequeira R.P., Edwards A.M., Clarke T.B. (2023). Symbiotic firmicutes establish mutualism with the host via innate tolerance and resistance to control systemic immunity. Cell Host Microbe..

[bib0032] Karangiya V.K., Savsani H..H., Patil S.S., Garg D.D., Murthy K.S., Ribadiya N.K., Vekariya S.J. (2016). Effect of dietary supplementation of garlic, ginger and their combination on feed intake, growth performance and economics in commercial broilers. Vet. World..

[bib0033] Kouvedaki I., Pappas A.C., Surai P.F., Zoidis E. (2024). Nutrigenomics of natural antioxidants in broilers. Antioxidants..

[bib0034] Lee K.W., Everts H.., Kappert H.J., Frehner M., Losa R., Beynen A.C. (2003). Effects of dietary essential oil components on growth performance, digestive enzymes and lipid metabolism in female broiler chickens. Br. Poult. Sci..

[bib0035] Li S., Ren L., Zhu X., Li J., Zhang L., Wang X., Gao F., Zhou G. (2019). Immunomodulatory effect of γ-irradiated astragalus polysaccharides on immunosuppressed broilers. Anim. Sci. J..

[bib0036] Li Y., Xu B., Xu M., Chen D., Xiong Y., Lian M., Sun Y., Tang Z., Wang L., Jiang C., Lin Y. (2017). 6-Gingerol protects intestinal barrier from ischemia/reperfusion-induced damage via inhibition of p38 MAPK to NF-kappaB signalling. Pharmacol. Res..

[bib0037] Liu M., Ma J., Xu J., Huangfu W., Zhang Y., Ali Q., Liu B., Li D., Cui Y., Wang Z., Sun H., Zhu X., Ma S., Shi Y. (2024). Fecal microbiota transplantation alleviates intestinal inflammatory diarrhea caused by oxidative stress and pyroptosis via reducing gut microbiota-derived lipopolysaccharides. Int. J. Biol. Macromol..

[bib0038] Long X., Zhang F., Wang L., Wang Z. (2024). The chicken cecal microbiome alters bile acids and riboflavin metabolism that correlate with intramuscular fat content. Front. Microbiol..

[bib0039] Lopez-Siles M., Duncan S.H., Garcia-Gil L.J., Martinez-Medina M. (2017). Faecalibacterium prausnitzii: from microbiology to diagnostics and prognostics. ISME J..

[bib0040] Mason J.R., Bean N..J., Shah P.S., Clark L. (1991). Taxon-specific differences in responsiveness to capsaicin and several analogues: correlates between chemical structure and behavioral aversiveness. J. Chem. Ecol..

[bib0041] Miquel S., Martín R., Rossi O., Bermúdez-Humarán L.G., Chatel J.M., Sokol H., Thomas M., Wells J.M., Langella P. (2013). Faecalibacterium prausnitzii and human intestinal health. Curr. Opin. Microbiol..

[bib0042] Mir N.A., Rafiq A.., Kumar F., Singh V., Shukla V. (2017). Determinants of broiler chicken meat quality and factors affecting them: a review. J. Food Sci. Technol..

[bib0043] Miyamoto M., Matsuzaki K., Katakura M., Hara T., Tanabe Y., Shido O. (2015). Oral intake of encapsulated dried ginger root powder hardly affects human thermoregulatory function, but appears to facilitate fat utilization. Int. J. Biometeorol..

[bib0044] Niknia A.D., Vakili R.., Tahmasbi A.M. (2023). Role of zinc-methionine chelate on bone health and eggshell quality in late–phase laying hens. All Life..

[bib0045] Owuor E.D., Kong A.N. (2002). Antioxidants and oxidants regulated signal transduction pathways. Biochem. Pharmacol..

[bib0046] Pan D., Yu Z. (2014). Intestinal microbiome of poultry and its interaction with host and diet. Gut Microbes..

[bib0047] Qiao H., Zhao T., Yin J., Zhang Y., Ran H., Chen S., Wu Z., Zhang R., Wang X., Gan L., Wang J. (2022). Structural characteristics of inulin and microcrystalline cellulose and their effect on ameliorating colitis and altering colonic microbiota in dextran sodium sulfate-induced colitic mice. ACS Omega.

[bib0048] Schloss P.D., Westcott S..L., Ryabin T., Hall J.R., Hartmann M., Hollister E.B., Lesniewski R.A., Oakley B.B., Parks D.H., Robinson C.J., Sahl J.W., Stres B., Thallinger G.G., Van Horn D.J., Weber C.F. (2009). Introducing mothur: open-source, platform-independent, community-supported software for describing and comparing microbial communities. Appl. Environ. Microbiol..

[bib0049] Shanmugavelu S., Acamovic T., Cowieson A.J. (2004). Effect of thyme oil and garlic powder on the nutritive value of soybean meal. Br. Poult. Sci..

[bib0050] Shanoon A., Amin Q., Ezaddin I. (2012). Effects of ginger (Zingiber officinale) oil on growth performance and microbial population of broiler Ross 308. Int. J. Poult. Sci..

[bib0051] Shewita R.S., Taha A.E. (2018). Influence of dietary supplementation of ginger powder at different levels on growth performance, haematological profiles, slaughter traits and gut morphometry of broiler chickens. S. Afr. J. Anim. Sci..

[bib0052] Shin J.H., Tillotson G.., MacKenzie T.N., Warren C.A., Wexler H.M., Goldstein E.J.C. (2024). Bacteroides and related species: the keystone taxa of the human gut microbiota. Anaerobe..

[bib0053] Stubhaug T.T., Giske C..G., Justesen U.S., Kahlmeter G., Matuschek E., Sundsfjord A., Skaare D. (2023). Antimicrobial susceptibility testing of Bacteroides species by disk diffusion: the NordicAST Bacteroides study. Anaerobe..

[bib0054] Su Y., Huang P., Wu Z., Dai W., Zhang Y., Zeng J. (2024). Effect of dietary supplementation with sanguinarine on meat quality and lipid metabolism of broilers. Poult. Sci..

[bib0055] Sumanu V.O., Naidoo V.., Oosthuizen M.C., Chamunorwa J.P. (2022). Adverse effects of heat stress during summer on broiler chickens production and antioxidant mitigating effects. Int. J. Biometeorol..

[bib0056] Szolcsányi J., Sann H., Pierau F.K. (1986). Nociception in pigeons is not impaired by capsaicin. Pain..

[bib0057] Talukder S., Hasan M.M., Noman Z.A., Sarker Y.A., Paul T.K., Sikder M.H. (2017). Effect of dietary supplementation of ginger extract on growth, carcass characteristics and haematological parameters in broilers. Asian J. Med. Biol. Res..

[bib0058] Tekeli A., Kutlu H., Baykal Çelik L., D F. (2010). Determination of the effects of Z. officinale and propolis extracts on intestinal microbiology and histological characteristics in broilers. Int. J. Poult. Sci..

[bib0059] Vakili R., Mokhtarpour A., Hosseini Ghaffari S.A. (2025). Functional feed for laying hens: application of saffron extract as eco-friendly supplement with cholesterol-lowering properties. Vet. Med. Sci..

[bib0060] Vakili R., Rashidi A.A. (2011). The effects of dietary fat, vitamin E and zinc supplementation on fatty acid composition and oxidative stability of muscle thigh in broilers under heat stress. Afr. J. Agric. Res..

[bib0061] Vakili R., Zanghaneh A., Qharari F., Mortzavi F. (2025). Hydroalcoholic extract of saffron petals, yeast cell wall and bentonite reduce contamination effects with aflatoxin B1 and ochratoxin A in exposed broilers. Vet. Med. Sci..

[bib0062] Wen C., Liu Y., Ye Y., Tao Z., Cheng Z., Wang T., Zhou Y. (2020). Effects of gingerols-rich extract of ginger on growth performance, serum metabolites, meat quality and antioxidant activity of heat-stressed broilers. J. Therm. Biol..

[bib0063] Wisdom A., Ikwunze K., Oguike M., Onunkwo D. (2018). Impact of ginger (Zingiber officinale) on intestinal, caeca microbial loads and growth performance of broilers. Niger. J. Anim. Sci..

[bib0064] Yang K., Lu Y., Yue Z., Jin S., Wang P., Liu C., Wang L., Yin Q., Dang X., Guo H., Chang J. (2024). 6-Gingerol activates the Nrf2 signaling pathway to alleviate hydrogen peroxide induced oxidative stress on primary chicken embryo hepatocytes. J. Funct. Foods.

[bib0065] Zhang X.L., Chen L.., Yang J., Zhao S.S., Jin S., Ao N., Yang J., Liu H.X., Du J. (2023). Vitamin D alleviates non-alcoholic fatty liver disease via restoring gut microbiota and metabolism. Front. Microbiol..

