# Supplementary material for: Effects of ginger ethanol extract on growth performance, antioxidant capacity and intestinal microbiota of broilers
Source: Poult Sci. 2025 Nov 20;105(1):106139. doi: 10.1016/j.psj.2025.106139 (PMC12723038; doi:10.1016/j.psj.2025.106139)
Supplement: Supplementary file 1 [file mmc1.docx]

**Table S1.** Gingerol elution gradient procedures

| Time (min) | Mobile phase A (%) | Mobile phase B (%) |
| --- | --- | --- |
| 0-10 | 45 | 55 |
| 10-15 | 45→48 | 55→52 |
| 15-17 | 48→60 | 52→40 |
| 17-43 | 60 | 40 |
| 43-45 | 60→67 | 40→33 |
| 45-48 | 67→69 | 33→31 |
| 48-58 | 69→71 | 31→29 |

^1^ Each value represents the mean value of 6 samples (n=6)

**Table S2.** Effect of different ethanol concentrations on the extraction of 6-gingerol

| Groups | Content (%) |
| --- | --- |
| 70% | 1.10±0.03^b^ |
| 75% | 1.23±0.01^a^ |
| 80% | 1.20±0.02^ab^ |
| 85% | 1.22±0.04^a^ |
| 90% | 1.19±0.12^ab^ |
| 95% | 1.18±0.03^ab^ |
| 99% | 1.26±0.05^a^ |

^a, b^ Means with no common superscript within a column differ significantly (*P* < 0.05)

^1^ Each value represents the mean value of 6 samples (n=6)

**Table S3.** The effect of different liquid-solid ratio on the extraction of 6-gingerol

| Groups | Content (%) |
| --- | --- |
| 6 mL/g | 0.82±0.02^c^ |
| 7 mL/g | 0.85±0.01^c^ |
| 8 mL/g | 0.94±0.04^a^ |
| 9 mL/g | 0.90±0.01^b^ |
| 10 mL/g | 0.92±0.01^ab^ |

^a, b^ Means with no common superscript within a column differ significantly (*P* < 0.05)

^1^ Each value represents the mean value of 6 samples (n=6)

**Table S4.** The effect of different extraction time on the extraction of 6-gingerol

| Groups | Content (%) |
| --- | --- |
| 0.5 h | 0.87±0.07^b^ |
| 1.0 h | 0.99±0.03^a^ |
| 1.5 h | 0.88±0.01^b^ |
| 2.0 h | 0.86±0.02^b^ |
| 2.5 h | 0.84±0.03^b^ |

^a, b^ Means with no common superscript within a column differ significantly (*P* < 0.05)

^1^ Each value represents the mean value of 6 samples (n=6)

**Table S5.** Effect of different extraction temperatures on the extraction of 6-gingerol

| Groups | Content (%) |
| --- | --- |
| 20℃ | 0.90±0.07 |
| 30℃ | 0.88±0.05 |
| 40℃ | 0.95±0.01 |
| 50℃ | 0.95±0.14 |
| 60℃ | 0.85±0.04 |

^a, b^ Means with no common superscript within a column differ significantly (*P* < 0.05)

^1^ Each value represents the mean value of 6 samples (n=6)

**Table S6.** Analysis of the ginger active components

| Ingredient | Content (mg/g) |
| --- | --- |
| 6-gingerol | 10.01 |
| 8-gingerol | 1.57 |
| 10-gingerol | 1.60 |

^1^ Each value represents the mean value of 6 samples (n=6)
